# Thermosensitive Polymeric Nanoparticles for Drug Co-Encapsulation and Breast Cancer Treatment

**DOI:** 10.3390/pharmaceutics16020231

**Published:** 2024-02-05

**Authors:** Vanessa Franco Carvalho Dartora, Julia S. Passos, Leticia V. Costa-Lotufo, Luciana B. Lopes, Alyssa Panitch

**Affiliations:** 1 Department of Pharmacology, Institute of Biomedical Sciences, University of São Paulo (USP), São Paulo 05508-900, Brazil; vanessa.dartora@gatech.edu (V.F.C.D.); julia.sapienza.passos@usp.br (J.S.P.); costalotufo@gmail.com (L.V.C.-L.); lublopes@usp.br (L.B.L.); 2 Department of Biomedical Engineering, College of Engineering, University of California Davis, Davis, CA 95616, USA; 3 Wallace H. Coulter Department of Biomedical Engineering, College of Engineering, Georgia Institute of Technology, School of Medicine, Emory University, Atlanta, GA 30322, USA

**Keywords:** ductal carcinoma in situ, intraductal administration, piplartine, piperlongumine, PNIPAm, nanoparticle, MK2 inhibitor

## Abstract

Despite advances in breast cancer treatment, there remains a need for local management of noninvasive, low-grade ductal carcinoma in situ (DCIS). These focal lesions are well suited for local intraductal treatment. Intraductal administration supported target site drug retention, improved efficacy, and reduced systemic exposure. Here, we used a poly(N-isopropyl acrylamide, pNIPAM) nanoparticle delivery system loaded with cytotoxic piplartine and an MAPKAP Kinase 2 inhibitor (YARA) for this purpose. For tumor environment targeting, a collagen-binding peptide SILY (RRANAALKAGELYKSILYGSG-hydrazide) was attached to pNIPAM nanoparticles, and the nanoparticle diameter, zeta potential, drug loading, and release were assessed. The system was evaluated for cytotoxicity in a 2D cell culture and 3D spheroids. In vivo efficacy was evaluated using a chemical carcinogenesis model in female Sprague–Dawley rats. Nanoparticle delivery significantly reduced the IC_50_ of piplartine (4.9 times) compared to the drug in solution. The combination of piplartine and YARA in nanoparticles further reduced the piplartine IC_50_ (~15 times). Treatment with these nanoparticles decreased the in vivo tumor incidence (5.2 times). Notably, the concentration of piplartine in mammary glands treated with nanoparticles (35.3 ± 22.4 μg/mL) was substantially higher than in plasma (0.7 ± 0.05 μg/mL), demonstrating targeted drug retention. These results indicate that our nanocarrier system effectively reduced tumor development with low systemic exposure.

## 1. Introduction

The majority of breast cancer lesions begin in the lining of the mammary duct [1,2], and among its many forms, ductal carcinoma in situ (DCIS) makes up approximately 20–25% of diagnosed breast cancer types [3,4]. Atypical lesions, such as atypical ductal hyperplasia (ADH), and benign lesions (such as multiple intraductal papillomas) may progress to ductal carcinoma in situ (DCIS) and to invasive cancer. Therefore, treatment is important to stop these lesions from developing into invasive cancer forms [5,6,7].

Intraductal therapy, in which a drug is administered directly into the mammary ducts through the nipple, is a promising approach for treating atypical lesions and DCIS [8]. Providing local intraductal intervention may reduce systemic side effects while treating the tumor itself [9]. The feasibility of this delivery route has been demonstrated for various drugs, including paclitaxel, doxorubicin, curcumin, and carboplatin, in animal models with chemically induced carcinomas [10,11,12,13,14]. The delivery route has also been demonstrated to be safe and well tolerated in clinical settings [9,15], where a significant reduction in tumor burden, without significant systemic adverse effects, was observed [16].

Taking into consideration the characteristics and goals of the intraductal route, nanocarriers, such as nanoemulsions, lipid nanoparticles, polymeric aggregates, and nanosuspensions, have been proposed as delivery systems to prolong mammary tissue retention and favor local drug effects over potential system toxic effects by reducing systemic delivery while maintaining local therapeutically active doses [17,18,19,20,21,22]. In this study, stimuli-responsive polymeric nanoparticles for collagen targeting and intraductal delivery of the cytotoxic agents were used to load a new drug combination and evaluated for their effectiveness against 2D, 3D, and in vivo breast cancer models.

Stimuli-responsive polymeric nanoparticles have been widely explored for various applications related to cancer therapy. These polymers respond to small external changes in their environment with dramatic property variations. Examples of stimuli that promote these changes include temperature, pH, ionic strength, light, and electric or magnetic field [23,24]. While the pH of the systemic circulation is approximately 7.4, the pH of solid tumors is more acidic (between 5.7 and 7.2); thus, nanoparticles that collapse in acidic pH favor drug release in the tumor system while disfavoring it systemically [24]. Furthermore, the tumor microenvironment often presents higher temperatures (over 40 °C) than healthy tissues due to its greater metabolism rate, which also justifies the temperature-triggered drug delivery strategy for the mammary tumoral tissue [25]. Poly(N-isopropylacrylamide) (pNIPAM) is one of the most studied thermally actuating polymers, with thermoreversible gelation properties in aqueous solutions. Crosslinked pNIPAM forms a polymer-rich gel at temperatures in the range of 32–35 °C and swells to form an expanded network upon cooling [23,26]. This supports drug loading when the polymer is below 32 °C, and at physiological temperatures pNIPAM collapses, allowing pNIPAM nanoparticles to entrap a drug and slowly release it while simultaneously protecting the drug from enzymes and physical–chemical degradation [27,28].

In the present work, pNIPAM was copolymerized with the monomers acrylic acid (AAc) and 2-acrylamido-2-methyl-1-propanesulfonic acid (AMPS) [29,30]. Acrylic acid was added to provide carboxylic groups and to facilitate the functionalization of the nanoparticles; its concentration was set at 1 mol% aiming for an LCST of approximately 34 °C, because an increase in concentration could result in an LCST above the physiological temperature impairing drug release [29,31]. AMPS was added to provide negative charges to the particle to both promote the electrostatic interaction of the cationic peptides with the particle, and to support colloidal stability [29]. The degradable crosslinker N,N′-bis(acryloyl)cystamin (BAC) was also added to the particle, seeking to facilitate its degradation and to assist in the sustained release of active ingredients [30]. Previously, we showed that increasing the crosslink density for pNIPAM nanoparticles affects swelling and decreases the particle diameter [30]. Additionally, the crosslink density affects drug loading and release. Overall, the monomer composition was designed with particle stability, drug loading and release, and particle degradation in mind.

In this study, piplartine and the positively charged MAPKAP Kinase 2 (MK2) inhibiting peptide YARAAARQARAKALARQLGVAA (abbreviated YARA) were co-encapsulated in collagen-binding pNIPAM nanoparticles to potentiate cytotoxicity. Piplartine is a cytotoxic and antiproliferative alkaloid/amide component of the *Piper* species, and may lead to cell death both by apoptosis and necrosis, depending on the concentration range employed [32,33,34]. Previous reports demonstrated that blocking MK2 activity makes p53 mutant tumor cells more vulnerable to chemotherapy [35,36,37]. An in vivo study demonstrated that tumors lacking both p53 and MK2 shrank dramatically when treated with the drug cisplatin, while tumors with functional MK2 kept growing even after treatment [38]. Because approximately half of cancer patients have a mutation in p53, which mediates survival, a combination of piplartine and an MK2 inhibitor to enhance the sensitization of tumors is a promising approach [39]. Furthermore, because a variety of tumors over-express collagen, which constitutes the physical scaffold of the tumor microenvironment, pNIPAM nanoparticles were modified with the collagen-binding peptide-hydrazide SILY (RRANAALKAGELYKSILYGSG-hydrazide).

In the first part of this study, the physicochemical properties of NPs, including size, polydispersity index (PDI), ζ potential, drug loading and release, and collagen-binding ability, were evaluated. Having defined the composition, selected nanoparticles were evaluated for uptake and cytotoxicity against breast cancer cells in 2D cultures and 3D spheroids, and an in vivo proof of concept was conducted in a chemically induced carcinogenesis model. We believe that the results presented here support the benefits of the combination of collagen-binding nanoparticles, MK2i peptide and piplartine co-encapsulation, and the intraductal route as a promising new strategy for potential applicability in local breast cancer management.

## 2. Materials and Methods

### 2.1. Material

N-isopropylacrylamide (≥98%, NIPAm) was acquired from Polysciences Inc. (Warrington, PA, USA). Dialysis membrane tubing was purchased from Spectrum Laboratories (Dominguez, CA, USA). Absolute ethanol (100%), sodium dodecyl sulfate (SDS; 10% *w*/*v* in water), 2-acrylamido-2-methyl-1-propanesulfonic acid (99%, AMPS), dithiothreitol (98%, DTT), N,N-diisopropylethylamine (99%, DIPEA), potassium persulfate (99%, K2S2O8), N,N’-bis(acryloyl)cystamine (98%, BAC), and dimethyl sulfoxide (DMSO) were acquired from Sigma-Aldrich (St. Louis, MO, USA). NIPAm, BAC, and AMPS were stored under nitrogen at 4 °C. All water used in the synthesis, dialysis, and testing was treated by a Milli-Q system (Millipore, Billerica, MA, USA; 18.2 MΩ·cm resistivity).

### 2.2. Peptide Synthesis

The peptides YARA (YARAAARQARAKALARQLGVAA, molecular weight: 2283.6 kDa) and hydrazide-SILY (RRANAALKAGELYKSILYGC, molecular weight: 2252.6 kDa) were synthesized on Rink-amide resin (CEM Corp.) or Cl-TCP(Cl) ProTide (CEM Corp.), respectively, on a Liberty Blue™ Peptide Synthesizer (CEM Corp., Matthews, NC, USA) using an FMOC (9-fluorenylmethyloxycarbonyl) solid-phase methodology [40,41]. Following synthesis, peptides were cleaved from the resin with 95% trifluoroacetic acid, 2.5% water, and 2.5% triisopropylsilane; precipitated in cold diethyl ether; and collected by centrifugation [40,41]. The recovered peptides were dried in vacuo, resuspended in Milli-Q water, and purified on an FPLC (ÄKTA Explorer, GE Healthcare, Salt Lake City, UT, USA) equipped with a 22/250 C18 prep-scale column (Aapptec, Louisville, KY, USA) using an acetonitrile gradient with a constant concentration of 0.1% trifluoroacetic acid. The molecular weight was confirmed by time-of-flight MALDI mass spectrometry using a 4800 Plus MALDI TOF/TOF™ Analyzer (Applied Biosystems, Foster City, CA, USA). Fractions containing purified peptides were lyophilized and stored at −80 °C.

### 2.3. Nanoparticle Generation

Hollow NIPAm-based nanoparticles (NPs), with 2-acrylamido-2-methyl-1-propanesulfonic acid (AMPS) and acrylic acid (AAc) were synthesized using a standard precipitation polymerization reaction [42,43,44]. For core synthesis, 30 mL of Milli-Q water was heated to 70 °C in a 3-neck round bottom flask and refluxed under nitrogen for 30 min. Subsequently, 397.4 mg of NIPAm and 164 μL of SDS were dissolved in 5 mL of Milli-Q water and added to the flask. Finally, 67.4 mg of potassium persulfate was dissolved in 2 mL of Milli-Q water and added to the flask to initiate the reaction. The polymerization was carried out for 2 h under nitrogen. To create the pNIPAM shell, 794.5 mg of NIPAm, 1 mol% AAc, 5 mol% AMPS and 164 μL of SDS were dissolved in 5 mL of Milli-Q water and added to the flask. A separate solution of 24.1 mg BAC (disulfide crosslinker) was prepared in 10 mL of Milli-Q water and added to the flask. To initiate the polymerization, 33.7 mg of KPS was dissolved in 2 mL of Milli-Q water and added to the flask. After 4 h, the core-shell nanoparticles were collected, cooled at room temperature and dialyzed against Milli-Q water for 7 days using a 15 kDa MWCO dialysis membrane Spectra-Por (Repligen, Waltham, MA, USA). Following dialysis, the particles were lyophilized and stored at room temperature. For fluorescent nanoparticle synthesis (NP-Rhodamine), 1 mol% rhodamine B isothiocyanate (98%, RBITC) was predissolved in 3% DMSO in Milli-Q water and then added to the shell polymer mixture (NIPAm, AMPS, BAC, AAc, and SDS) and allowed to equilibrate for 30 min before the polymerization reaction was initiated with the addition of potassium persulfate. This resulted in the formation of fluorescently labeled co-poly(NIPAm-AMPS-AAc-BAC-RBITC) shells surrounding the core. The following steps proceeded as described above. All samples were kept in the dark for further experiments.

### 2.4. SILY Peptide Attachment

Collagen-binding nanoparticles were obtained by using 1-ethyl-3-(3-dimethylaminopropyl) carbodiimide hydrochloride (EDC) to react the free carboxyl groups present on the nanoparticles with the hydrazide peptide [43]. First, 10 mg of lyophilized poly(NIPAm-AMPS-AAc) nanoparticles were activated by dissolving at 5 mg/mL in a coupling buffer consisting of 0.1 M MES (Sigma-Aldrich), 8 M Urea (Sigma-Aldrich) and 1 mg/mL EDC (Thermo Fisher Scientific, Waltham, MA, USA) at pH 4.5. After 15 min, a 1:2 or 1:1 molar equivalent of hydrazide SILY (SILY:AAc, which will also be referred to as NP 50 and 100%) was then added to the solution and allowed to shake at room temperature for 3 h. For collagen-binding affinity assay, 1% of the total concentration of the collagen-binding peptide was added as SILY_biotin_ and allowed to shake at room temperature for 1 h. The remaining peptide was added and allowed to react at room temperature for additional 2 h. The SILY conjugated nanoparticles (NP-SILY) were purified by centrifugation at 18,000× *g* and 25 °C for 30 min. The pelleted particles were rinsed with Milli-Q water, lyophilized and stored at room temperature [29,43]. Coupling efficiency of SILY to the nanoparticle was confirmed spectroscopically at 280 nm measuring the aromatic residues (Tyr) using a NanoDrop system.

### 2.5. NP-SILY Characterization

Size and Zeta (ζ) potentials were measured using a Nano-ZS90 Zetasizer equipment (Malvern, Westborough, MA, USA). Transmission electron microscopy (TEM, UC Davis School of Medicine, FEI CM120, Hillsboro, OR, USA) was used to investigate the nanoparticles structure and morphological aspect. First, a 7 μL droplet of MilliQ water containing suspended nanoparticles were placed in discharged TEM grids for 10 min. Then, samples were stained with uranyl acetate, dried, and imaged at room temperature. Confirmation of nanoparticle degradation was performed using hollow nanoparticles labeled with 1 mol% rhodamine B isothiocyanate. Degradation studies were performed in PBS pH 7.4 and PBS pH 3.5. The absorbance of the hollow nanoparticles dispersed at a final concentration of 1 mg/mL was monitored over a period of 7 days using a Spectramax M5 plate reader (Molecular Devices, Sunnyvale, CA, USA) at 544 nm.

### 2.6. NP-SILY In Vitro Collagen-Binding Assay

In order to verify the collagen-binding affinity of the modified nanoparticles (NP-SILY), the streptavidin-HRP colorimetric assay was used to detect the presence of the biotinylated particles on a collagen-coated surface [29,43]. NP 50 and 100% were evaluated, representing 1:2 and 1:1 molar equivalent of SILY/AAc, respectively. First, collagen-coated 96-well plates (Corning Biocoat, VWR) were blocked with 1% bovine serum albumin (BSA, VWR) in 1× PBS (200 μL) for 1 h at room temperature. Modified nanoparticles were dissolved in 1% BSA in 1× PBS at concentrations between 0 and 1 mg/mL. The blocking solution was removed, the nanoparticle solutions were added in triplicate to the plate and incubated on a plate shaker at 200 RPM and 37 °C for 30 min. The plate was rinsed with 1× PBS three times. Then, a diluted streptavidin-HRP solution (100 μL) was added to each well and incubated on a plate shaker at 200 RPM and room temperature for 20 min. The streptavidin-HRP solution was removed, the plate was rinsed three times with 1× PBS and 100 μL of a reagent color solution (R & D Systems, Minneapolis, MN, USA) was added to each well and allowed to incubate at 200 RPM and room temperature for 20 min. Finally, 50 μL of 2 N H_2_SO_4_ was added to stop the reaction, and the absorbance was read at 450 and 540 nm on a SpectraMax M5 plate reader.

### 2.7. Drug Loading and Release from NP-SILY

The loading of the drugs into the lyophilized hollow NIPAm SILY-modified nanoparticles was performed by swelling, and 1 mg of NP-SILY, 2 mg of the peptide YARA, and 2 mg of piplartine were co-incubated in 1 mL of 100% ethanol (Sigma-Aldrich) and stored at 4 °C for 24 h [43]. The loaded particles (NP-SILY-PIP-YARA) were then pelleted via centrifugation at 18,000× *g* and 25 °C for 30 min, and the supernatant was collected for fluorescence or chromatography analysis. The precipitate was resuspended in 1 mL of Milli-Q water before being frozen and lyophilized for storage. The drug release was measured by resuspending loaded nanoparticles in 1 mL of 1× PBS, and the particles were incubated on a plate shaker at 37 °C and 200 RPM. At predefined time points, the nanoparticles were pelleted via centrifugation, and the supernatant was collected. The pellet was then resuspended in 1× PBS and placed back on the plate shaker.

The fluoraldehyde o-phthalaldehyde (OPA) assay using 96-well, black, and flat-bottom plates (VWR) was performed to determine the amount of YARA loaded into and released by the nanoparticles. Samples (20 μL/well) were tested in triplicate on each plate by the addition of 200 μL of OPA reagent. The plates were incubated in the dark for 5 min, and the fluorescence was measured at an excitation of 360 nm and emission of 455 nm using a SpectraMax M5 plate reader [31]. The values were compared against a standard curve to determine the amount of YARA present in each solution.

Chromatographic analyses were performed to quantify the amount of piplartine loaded into and released by the nanoparticles using a Shimadzu HPLC system equipped with a pump (model LC-20AB), an autosampler (model SIL-20A), a UV detector (model SPD-M20A) set at 325 nm, and LabSolutions CS software (Shimadzu, Columbia, MD, USA). Piplartine separation was performed according to a previously developed and validated method in a Phenomenex C18 (150 mm × 4.6 mm) column maintained at 25 °C [17,45]. The mobile phase was composed of 1:1 (*v*/*v*) acetonitrile:water (pH adjusted to 4.0 with 0.1% acetic acid) and used at a flow rate of 1 mL/min. The injected volume into the column was 20 μL for the samples and calibration curve. The calibration curve was prepared in methanol and used to determine the amount of piplartine present in each solution. For each experiment, new calibration curves were obtained. The drug loading efficiency was then determined by measuring the amount of peptide or piplartine loaded per milligram of nanoparticle.

### 2.8. In Vitro Nanoparticle Efficacy and Uptake in Cells and Spheroid

MCF-7 and T47-D (breast cancer cells) were obtained from ATCC and cultured in DMEM GlutaMAX™ (Gibco, Carlsbad, CA, USA) medium supplemented with 10% fetal bovine serum (FBS, Gibco, Carlsbad, CA, USA) and penicillin and streptomycin (100 U/mL and 100 μg/mL Gibco, Carlsbad, CA, USA). Cells were grown at 37 °C and in a 5% CO_2_ atmosphere, and passages were performed at 80% confluence.

#### 2.8.1. Cytotoxicity of Nanoparticles in Cell Monolayers

Cytotoxicity assays were conducted with three goals in mind: first, to determine whether unloaded NP-SILY nanoparticles and YARA-loaded NP-SILY nanoparticles exhibit a cytotoxic effect against cancer cells; second, to assess the effect of nanoencapsulation on piplartine cytotoxicity against cancer cells; and, third, to study whether the association of piplartine and YARA in the NP-SILY was more cytotoxic than the individual drugs. A colorimetric MTS assay (CellTiter 96^®^, Promega, Madison, WI, USA) was performed to access cell viability after treatment. The cells were seeded in 96-well plates at 10,000 cells/well in DMEM—GlutaMAX™ culture medium without FBS and incubated at 37 °C in 5% CO_2_ for 24 h. Then, the cells were treated for 72 h with the unloaded nanoparticles (0.12–100 μg/mL), NP-SILY-YARA (YARA 0.1–35 μM), NP-SILY-PIP (piplartine 0.23–75 μM), or NP-SILY-PIP-YARA (YARA 0.1–35 μM and piplartine 0.23–75 μM) and with a piplartine solution in DMSO (0.23–75 μM). The DMSO-treated cells and cells treated with doxorubicin (0.02–9.2 μM) were used as negative and positive controls, respectively. For assessment of viability, 20 μL of CellTiter 96^®^ AQueous One Solution Reagent was added into each well containing the samples in 100 μL of culture medium. The cells were incubated for 3 h, and the soluble formazan produced by the cellular reduction of MTS was quantified using a SpectraMax M5 plate reader at 490 nm. The experiments were performed in triplicate and repeated 3–5 times. Average viability curves for each treatment group were fitted with polynomial models (using GraphPad Prism 8, GraphPad Software, San Diego, CA, USA) for estimation of the drug concentrations necessary to reduce cell viability to 50% (IC_50_).

#### 2.8.2. Cytotoxicity of Nanoparticles in Spheroids

To obtain MCF-7 and T47-D spheroids, the liquid overlay technique was used as previously described [46,47,48]. The cells were seeded at 5000 cells/well onto 96-well spheroid microplates, the plate was centrifuged at 142 RCF for 5 min and incubated at 37 °C for 5 days in a CO_2_ incubator. The formation of spheroids was confirmed by optical microscopy observation. Spheroids were exposed for 72 h to nanoparticles (1.17–150 μg/mL) containing piplartine (0.87–82 μM, NP-SILY-PIP) or containing the drug combination with the peptide YARA (0.96–123.7 μM, NP-SILY-PIP-YARA) and piplartine DMSO solution (0.87–112.5 μM). The DMSO-treated spheroids were used as a negative control. The spheroid viability was measured using a CellTiter-Glo Luminescent Cell viability assay (Promega, Madison, WI, USA).

#### 2.8.3. Assessment of Nanoparticle Uptake

A monolayer of MCF-7 and T47-D and spheroids of T47-D cultures were incubated for 48 h with Rhodamine B labeled nanoparticles (NP-SILY-Rhodamine) at 50 μg/mL (0.1 μM), DMSO Rhodamine B solution (0.1 μM), or DMSO as a negative control (untreated). Monolayer culture was washed 3 times with 1× PBS and fixed with 4% paraformaldehyde for 30 min. Nuclei were stained with 4,6-diamidino-2-phenylindole (DAPI; Sigma-Aldrich), and cells were visualized using confocal microscopy at 64× [47,49]. Before cryosectioning, spheroids were fixed for 30 min with 4% paraformaldehyde, washed 3 times with 1× PBS, and frozen at −80 °C in Tissue-Tek O.C.T. Samples were sectioned at a thickness of 10 μm using a cryo-microtome [50]. Then, fluorescent images were obtained to identify nanoparticles that target cells in the inner area of the tumor spheroid.

### 2.9. In Vitro Inhibition of MK2

To determine the effects of the MK2 inhibition pathway by treatment with nanoparticles containing YARA, we accessed the expression of phospho-Smad3 through immunohistochemistry after cell stimulation with TGF-β1 [51,52]. The cells were seeded in a black, 96-well plate with a flat and clear bottom (ibidi GmbH, Gräfelfing, Germany) at 10,000 cells/well in DMEM—GlutaMAX™ culture medium without FBS and incubated at 37 °C in 5% CO_2_ incubator for 24 h. Followed by PBS or 10 ng/mL TGF-β1 (PeproTech, Inc., Cranbury, NJ, USA) stimulation, the cells were treated with nanoparticles containing the peptide YARA (100 μg/mL corresponding to 35 μM of YARA) or a DMSO solution of YARA peptide (100 μg/mL corresponding to 35 μM of YARA) for 24 h. The DMSO-treated cells were used as a negative control. First, the cells were fixed with 4% paraformaldehyde for 20 min and permeabilized with Triton 0.5% in PBS 1 × and nonspecific staining blocked with bovine serum. Then, the cells were incubated with primary antibody against phospho-Smad3 Ser423/425 (Cell Signaling Technology, Inc., Danvers, MA, USA) overnight at 4 °C, followed by incubation with labeled anti-rabbit IgG (Alexa Fluor^®^ 555 Conjugate, Cell Signaling Technology, Inc.) for 2 h at room temperature. Nuclei were counterstained with 4,6-diamidino-2-phenylindole (DAPI; Sigma-Aldrich). Cells were visualized using a confocal fluorescent microscope at 64× magnification. In control experiments, no primary antibody was applied.

The inhibition of phosphorylation in the heat shock protein 27 (HSP27), which is a downstream substrate of MK2, was also studied. The cells were seeded in 96-well tissue culture plates at 10,000 cells/well and incubated at 37 °C and 5% CO_2_ for 24 h. Following the cells’ stimulation with 10 ng/mL IL-1 β and 0.4 ng/mL TGF-β1 (PeproTech, Inc., Cranbury, NJ, USA) for 1 h, the cell culture medium was replaced with the following treatments: fresh cell culture medium (positive control), NP-YARA (100 μg/mL corresponding to 35 μM of YARA), and YARA solution (35 μM). Poststimulation and treatment, the cells were subjected to a process of HSP27 extraction. This was followed by an ELISA (enzyme-linked immunosorbent assay) to detect phosphorylated HSP27 at serine 15 (Enzo Life Sciences, Inc., Farmingdale, NY, USA) in compliance with the instructions provided by the manufacturer. The experimental design included triplicate wells for each treatment type, and the entire procedure was replicated three times to ensure the reliability of the data.

### 2.10. Retention in Mammary Tissue

This experiment was designed to compare two aspects: (i) the capability of NPs to remain in mammary tissue and (ii) the benefits of using drug-loaded NPs over drug-based simple solutions for tissue targeting. Fluorescently labeled NPs were obtained according to Section 2.3. Fluorescence tracking in the animals was performed using whole-body imaging after intraductal administration. Control groups received blank NPs without the fluorescent marker. Female Wistar rats were kept under controlled conditions. They had free access to food and water and were maintained in an environment with a 12 h light–dark cycle with temperatures between 22 and 23 °C until they reached a weight of 250 ± 20 g. The experimental protocol adhered to the Brazilian Council for Control of Animal Experimentation (CONCEA) guidelines and was approved by the Animal Care and Use Committee of the Institute of Biomedical Sciences, University of São Paulo (Protocol number: 69/2016).

For the experiment, rats were anesthetized with isoflurane and prepared by removing abdominal hair [17,18]. After 24 h, the animals were grouped for different treatments: intraductal rhodamine solution, blank NPs, and rhodamine-labeled NPs; 4 animals/group were used. Under anesthesia, the rats received 10 μL of either treatment through their nipple ducts using a 33 G needle and Hamilton syringe [17,18]. The rhodamine fluorescence was monitored for 1 to 120 h using a whole-body bioimaging system (IVIS Spectrum System, Perkin-Elmer Life Sciences, Waltham, MA, USA). The following instrument settings were fixed for comparison among groups: exposure time = 5 s, binning factor = 8, and excitation/emission = 507/529 nm.

To evaluate the histological alterations potentially caused by the nanoparticles, mammary glands from animals subjected to blank NPs or rhodamine-labeled NPs were surgically removed 120 h after injection and compared with the untreated group. These excised tissues were then fixed using a 10% buffered neutral formaldehyde solution and subsequently prepared for paraffin embedding [11]. Histological sections, each measuring 5 μm in thickness, were generated and subsequently stained with hematoxylin/eosin before undergoing microscopic analysis. The investigation focused on the presence of tissue edema, infiltration of inflammatory cells, and changes in the morphology of ducts and lobular units [12].

### 2.11. In Vivo Proof of Concept of Nanoparticle Efficacy

Female Sprague–Dawley rats, aged 7–8 weeks, were kept in an environment with unlimited access to food and water. The room for the animals was set to a 12-h light–dark cycle, maintaining temperatures between 22 and 23 °C. The experimental procedures adhered to the standards set by the National Council for Animal Experimentation (CONCEA) and received approval from the Animal Care and Use Committee at the Institute of Biomedical Sciences, University of São Paulo, Brazil (protocol number: 70/2016). The experiments were carried out in the afternoon, between 1:00 p.m. and 5:00 p.m. To induce breast tumors, 1-methyl-1-nitrosourea (NMU; Spectrum Chemical MFG Corp., New Brunswick, NJ, USA) was used. The NMU was freshly dissolved in saline (0.9% NaCl) and adjusted to pH 4 with acetic acid [53]. The chosen dosage of 50 mg/kg was based on prior studies, which have demonstrated its efficacy in inducing mammary gland tumors while minimizing adverse effects [53,54].

This study was carried out in alignment with methodologies previously described [11,14,21]. Four weeks post-NMU administration, the subjects were allocated into 3 distinct groups (each comprising 8 animals). The first group, serving as the negative control (untreated, U), was administered saline with a pH of 4.0. The remaining groups (2 and 3) were given 50 mg/kg of NMU intraperitoneally to initiate tumor growth. Group 2, the positive control (C), received no subsequent treatment. Group 3 was treated with piplartine-loaded and YARA-loaded nanoparticles (NP-SILY-PIP-YARA). The free drug alone was not evaluated because of a lack of surrogate drug retention seen in the IVIS studies. Prior to treatment, the animals were anesthetized with isoflurane (2–2.5%), their abdominal hair removed using VEET^®^ cream, the surgical area sanitized with alcohol, and breast ducts accessed via a 33G needle attached to a 0.1 mL syringe [17,18]. Each mammary gland was intraductally injected with 20 μL of NP-SILY-PIP-YARA (1 mg/mL). This treatment was administered biweekly, totaling three sessions per animal. The criteria for early removal of animals in the study were defined as follows: reduction in body weight exceeding 30%, emergence of ulcers or skin lesions, growth in tumors to a maximum average size of 1.2 cm (for multiple tumors), and any alterations in behavior suggesting discomfort or pain [55].

A week after the final treatment, the animals were euthanized for mammary gland dissection. The glands were divided into two sets: One set was fixed in 10% neutral buffered formaldehyde for histological analysis, involving staining (hematoxylin–eosin) and examining epithelial hyperplasia or hypertrophy, proliferation/desquamation in ducts, hyperplastic nodules, and changes in surrounding connective tissue [56]. The second set underwent homogenization for piplartine level analysis using HPLC. Blood was also collected from the left ventricle to determine systemic piplartine levels. It should be noted that the objective of this study was not a comprehensive pharmacokinetic/pharmacodynamic (PK/PD) investigation but rather a comparison of piplartine levels in the plasma and mammary tissue after intraductal administration. For piplartine extraction and quantification, approximately 1 g of mammary tissue was processed in conical tubes containing 2 mL methanol. The tissue was homogenized using a Biospec products tissue homogenizer (Bartlesville, OK, USA), followed by vortexing for 30 s and sonication in a bath for 15 min [56]. The homogenate was then filtered through a PTFE membrane with a 0.45 μm pore size, and the piplartine content was determined using HPLC. The recovery rate of piplartine varied from 75 to 89% [21]. Blood samples were kept in BD SST^®^ II Advance^®^ Gel Tubes and subjected to centrifugation at 5000 RPM for 15 min to separate the plasma. Piplartine extraction involved mixing the plasma with an equal volume of acetonitrile, followed by centrifugation at 4000 rpm for 15 min and filtration before HPLC measurement (Section 2.7) [56]. Palpable tumors were excised, and their volume was calculated using a previously described formula [11]:

$$Tumor\;volume\;\left({mm}^{3}\right)=\frac{a\times b^{2}}{2}$$

where (*a*) and (*b*) are the longest and the shortest diameters, in mm, respectively.

### 2.12. Data Analysis

All results are expressed as the mean ± standard deviation. Statistical analysis was carried out using the computer software GraphPad Prism 8 (GraphPad Software, San Diego, CA, USA). The results were analyzed using paired and unpaired *t*-tests for comparisons between the two groups. For multiple comparisons, a mixed model of ANOVA was employed to compare the different treatment groups. Differences between the mean values of multiple groups were analyzed with a Tukey post hoc test. The differences were considered statistically significant at probability values of <0.05. Cell viability analyses were conducted with the same software by calculating the half maximum inhibitory concentration (IC_50_) and respective confidence intervals (95% CI) through nonlinear regression.

## 3. Results

### 3.1. Nanoparticle Characterization

**Figure 1:**
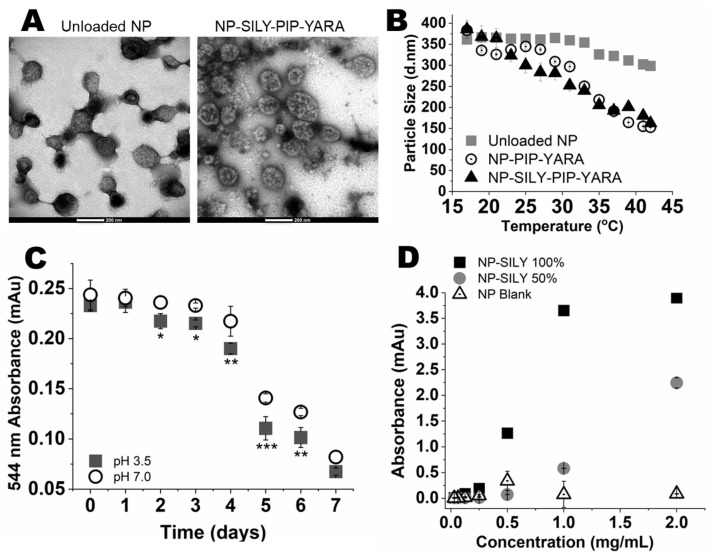
Characterization, stability, and collagen binding of pNIPAM nanoparticles modified with SILY: (**A**) TEM micrograph of unloaded and drug-loaded pNIPAM nanoparticles modified with SILY, scale bar = 200 nm. The nanoparticles were lyophilized and resuspended in Milli-Q water for 4 h at 25 °C prior to TEM. Scale bar = 200 nm. (**B**) Dynamic light scattering for the hydrodynamic diameter temperature sweep from 17 °C to 42 °C of unloaded NPs and NPs and NP-SILY loaded with piplartine and YARA. (**C**) Degradation of fluorescently labeled nanoparticles over 7 days of dialysis against 1× PBS pH 7.4 and 3.5 at 544 nm of absorbance. * *p* < 0.05, ** *p* < 0.001, and *** *p* < 0.0001 against pH 7.0. Data are the mean ± standard deviation of 6 replicates. (**D**) Collagen-binding assay demonstrating the ability of NP-SILY to bind to a collagen-I-coated surface. Particle binding increased with an increase in conjugated SILY, whereas blank nanoparticles did not show the ability to bind to the collagen plate.

In this study, thermosensitive pNIPAM-based nanoparticles modified with the collagen-binding peptide SILY were synthesized, and, following core removal, the nanoparticles underwent analysis encompassing size, surface charge, and shape, as depicted in Figure 1A and Table 1. Dynamic light scattering (DLS) analysis revealed that the nanoparticles retained their structural integrity after being freeze-dried and then rehydrated, with no significant change (*p* > 0.05) in particle size due to the encapsulation of the drug (Table 1). Additionally, transmission electron microscopy (TEM) provided visual evidence of the spherical particle shape postlyophilization and subsequent resuspension in Milli-Q water, as illustrated in Figure 1A. Collectively, this shows that the particles can be synthesized, loaded with drug, and lyophilized for storage.

As expected, the nanoparticles displayed a negative zeta potential as a result of integrating sulfated AMPS into their composition (Table 1) [57]. Following the conjugation with SILY, a shift toward more neutral zeta potential values was noted, attributable to the neutralizing effect of the amine groups in the SILY peptide on the surface charge of the nanoparticles. The change in zeta potential confirms SILY’s association with the nanoparticle surface [43].

Considering that pNIPAM is thermoresponsive, we next investigated whether SILY attachment or drug loading interfered with nanoparticle temperature responsiveness. DSL measurement of the nanoparticles’ hydrodynamic diameter as a function of temperature were conducted, using unloaded nanoparticles without SILY (unloaded NPs) as a control.

As can be observed in Figure 1, the lower critical solution temperature (LCST) was consistent with the literature (approximately 35 °C) [27,58,59]. A decrease in the hydrodynamic radius at temperatures above the LCST was observed for nanoparticles containing piplartine (PIP) and YARA (NP-PIP-YARA), with the radius decreasing by 31.2% when the particles collapsed at temperatures above the LCST. The ability of nanoparticles to respond to temperature was similar after SILY attachment (NP-SILY-PIP-YARA), maintaining a 32.3% reduction in the hydrodynamic radius at temperatures above the LCST.

Because of the importance of complete degradation for drug release, nanoparticle degradation as a function of time following exposure to pH 7.4 and 3.5 was investigated using hollow, rhodamine-labeled nanoparticles fabricated by direct polymerization of rhodamine isothiocyanate into the pNIPAM backbone; an absorbance sweep at 544 nm was conducted to monitor the rate of degradation over 7 days. Following incubation in PBS for 24 h, nanoparticles did not show significant signs of degradation, regardless of the pH (Figure 1C). After 48 h, degradation of nanoparticles was detected when they were exposed to pH 3.5. At pH 7.4, degradation was observed after 4 days. This suggests that the nanoparticles would not degrade or aggregate when stored at 25 °C for 4 days in Milli-Q water or PBS. Additionally, absorbance values were minimal after 7 days, with no significant difference between pH 3.5 and 7.4, suggesting complete nanoparticle degradation.

### 3.2. In Vitro Collagen Binding

The ability of SILY-modified nanoparticles to bind to a collagen-coated surface and the influence of the SILY ratio on binding were assessed using a streptavidin-HRP colorimetric assay [29,43]. As depicted in Figure 1D, the biotinylated SILY-modified nanoparticles were able to bind to type I collagen using 50% of the SILY content (as compared to acrylic acid content), demonstrating attachment at concentrations above 1 mg/mL. Increasing to 100% SILY resulted in an increase in collagen binding, with the NP-SILY binding to collagen observed at concentrations above 0.5 mg/mL. The nonmodified nanoparticles, used as a control, did not bind to the collagen-coated plate at concentrations up to 2 mg/mL, confirming that the curves obtained with NP-SILY are the result of the SILY peptide actively binding to collagen. The NP-SILY 100% was selected for further studies.

### 3.3. Drug Loading and Release

As demonstrated in Table 2, hollow nanoparticles successfully loaded 0.75 mg of YARA and 0.92 mg of piplartine, providing a final loading efficiency (as a percent of final particle weight) of 57.9% and 70.9%, respectively. The addition of SILY did not significantly (*p* = 0.228) interfere with piplartine loading (0.73 mg, 56.4%), but promoted a significant (*p* = 0.002) increase in YARA loading to 1.26 mg (97.1%).

**Figure 2:**
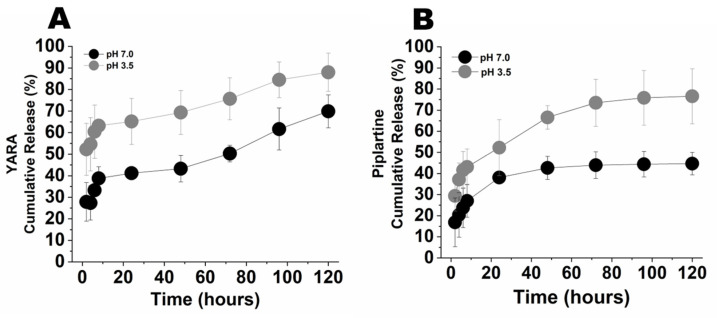
Release profiles of YARA (**A**) and piplartine (**B**) incorporated in NP-SILY in PBS at pH 3.5 and pH 7.4 over 120 h at 37 °C. Bars represent the average ± SEM (*n* = 12).

The release of piplartine and YARA from the nanoparticles after incubation in 1× PBS pH 7.4 and 3.5 at 37 °C is shown in Figure 2. A high initial burst release of piplartine and YARA from the nanoparticles was observed in both pH conditions, likely due to the rapid disassociation of drugs electrostatically bound to the nanoparticle’s outer shell. The amount of YARA and piplartine released at pH 7.4 was significantly lower than the amount released at pH 3.5. This variation was anticipated because of the more rapid reduction in disulfide bonds within the nanoparticles at the acidic pH, facilitating the release of the drugs [60]. Moreover, nanoparticle degradation also plays an integral role in the release process. At pH 3.5, substantial nanoparticle degradation was detected after 48 h, aligning with the rapid release of 86% of the YARA content within 120 h and half of that within the first 4 h (Figure 2A). In contrast, at pH 7.4, degradation commenced more slowly, becoming noticeable after 4 days, which corresponds with the more gradual release of 64% of the peptide over 120 h and 50% of this amount within the first 24 h. The particles showed a similar trend with piplartine (Figure 2B), releasing 80% of the drug at 120 h at pH 3.5 and 45% at pH 7.4, with 50% of the drug released within 4 h. Despite the rapid initial release rate, over 55% of the loaded piplartine remained in the nanoparticles after 120 h.

### 3.4. In Vitro Nanoparticle Effects on the Viability of Cell Monolayers and Spheroids

#### 3.4.1. MK2 Inhibition by YARA-Loaded Nanoparticles

**Figure 3:**
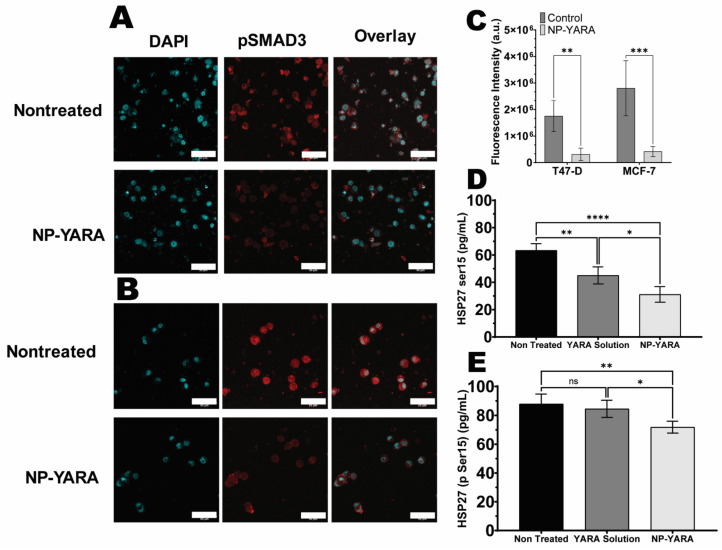
MCF-7 (**A**) and T47-D (**B**) cell staining for Smad3 (pSpS423/425). Cells were treated with 10 ng/mL TGF-beta for 1 h before treatment with the nanoparticles containing YARA (NP-YARA). Nontreated cells were used as a control. As a secondary antibody, Alexa Fluor^®^ 488 (Red) was used. Nuclei were stained with DAPI (Blue). The confocal images were captured at 60× magnification. Scale bar: 50 µm. (**C**) Quantification of the mean fluorescence intensity was by Fiji ImageJ2 software, and data are shown as the average ± standard deviation of 9 replicates in 3 independent experiments. Phosphorylation of HSP27 Serine 15 treated with YARA solution or NP-YARA for 48 h and compared with nontreated cells (positive control) in (**D**) MCF-7 and (**E**) T47-D cells. Error bars represent the standard deviation. * *p* < 0.05 ** *p* < 0.01, *** *p* < 0.001, and **** *p* < 0.0001, ns = non-significant compared to the control.

It has been shown that, even in their nonphosphorylated state, Smad2 and Smad3 are continually shuttling between the nucleus and cytoplasm states and that MAPK signaling regulates the subcellular distribution of pSmads in addition to regulating their phosphorylation [61,62]. To determine whether YARA released from the nanoparticles is active in vitro, we investigated the effect of nanoparticle treatment on the phosphorylation of Smad3 after TGF-β1 stimulation. As expected, TGF-β1 increased Smad3 phosphorylation, and a stronger fluorescent signal in both the controls of MCF-7 and T47-D cells was observed, which is consistent with normal Smad protein translocation (Figure 3A,B) [62]. In addition, a greater pSmad colocalization with nuclear staining is seen in the absence of YARA treatment (Figure 3A,B). As shown in Figure 3C, nanoparticle treatment following TGF-β1 stimulation reduced pSmad3 levels in both cell lines, suggesting the ability of nanoparticles to inhibit MK2-induced expression.

A study of HSP27 phosphorylation levels aimed to confirm that intracellular phosphorylation of HSP27 could be suppressed by YARA upon release from nanoparticles (NPs) in MCF-7 and T47-D cells [63]. As depicted in Figure 3D,E, treatment with YARA solution led to a decrease in phospho-HSP27 levels compared to the control (nontreated cells, *p* < 0.05) only in the MCF-7 cell line, confirming findings by Brugnano et al. (2011) [64]. Notably, treatment with NP-YARA led to a substantial reduction in phospho-HSP27 (statistical significance at *p* < 0.001) in both the MCF-7 and T47-D cells, with levels of approximately ~2.0 (MCF-7) and 1.2 (T47-D) times lower than those treated with YARA solution. These results imply that MK2i peptide therapy may effectively diminish TGF-β1-mediated phosphorylation of HSP27. Additionally, the presence of YARA in the NPs was observed to have this effect in both studied cell lines.

#### 3.4.2. Evaluation of Cell Viability (2D Model)

**Figure 4:**
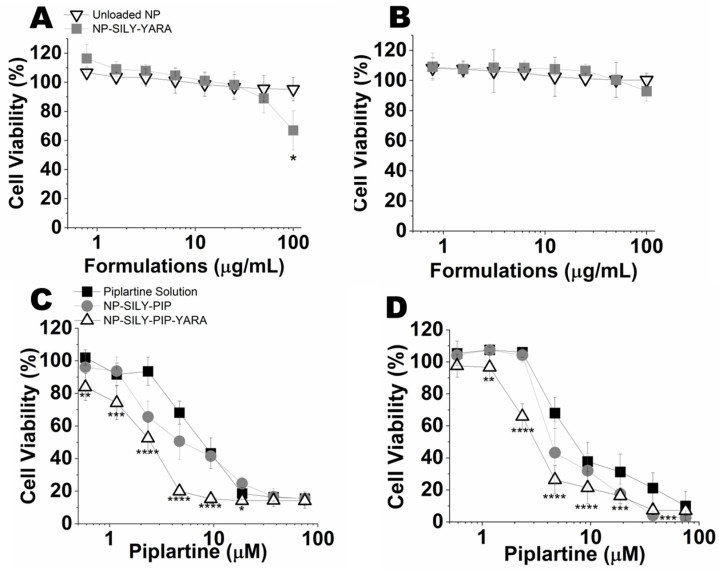
Viability of breast cancer cells after exposure to the unloaded nanoparticles and nanoparticles loaded with YARA, piplartine, or piplartine + YARA for 72 h: (**A**) comparison of the treatment of T47-D cells with unloaded (Unloaded NPs) or YARA-loaded nanoparticles (NP-SILY-YARA); (**B**) comparison of the treatment of MCF-7 cells with unloaded (Unloaded NPs) or YARA-loaded nanoparticles (NP-SILY-YARA); (**C**) comparison of the treatment of T47-D cells with piplartine solution and nanoparticles loaded with piplartine (NP-SILY-PIP) or piplartine+ YARA (NP-SILY-PIP_YARA); (**D**) comparison of the treatment of MCF-7 cells with piplartine solution and nanoparticles loaded with piplartine or piplartine + YARA. Data are shown as the average ± standard deviation of 10–15 replicates in 4–5 independent experiments. * *p* < 0.05, ** *p <* 0.01, *** *p <* 0.001 and **** *p <* 0.0001 compared to unloaded nanoparticles.

The cytotoxicity of unloaded nanoparticles and nanoparticles containing YARA was tested at concentrations up to 100 μg/mL. Unloaded NP-SILY did not reduce the cell viability in any of the cell lines at the concentration range studied (Figure 4). Only at the highest concentration tested (100 μg/mL, YARA at 82.5 μM), YARA-loaded NP-SILY reduced T47-D cell viability to 67% (Figure 4A); this effect was not observed in the MCF-7 cells (Figure 4B). This indicates that the particles are minimally cytotoxic at the concentration range studied.

Next, we evaluated whether the incorporation of drugs in the nanoparticles resulted in greater cytotoxicity compared to the drug in solution (Figure 4C,D and Table 3). The piplartine solution was cytotoxic in both lines tested, with a dose-dependent effect and IC_50_ values (50% inhibitory concentration) ranging from 9.5 µM to 10.6 µM for T47-D and MCF-7, respectively (Table 3). Compared to the solution, encapsulation of piplartine in NP-SILY promoted an increase in the cytotoxic effect, as observed by a shift of the viability curve to the left and 1.5–1.6 reductions in the IC_50_ values. The presence of YARA further decreased the IC_50_ values compared to piplartine in solution, and a reduction of 2.6-fold (4.0 μM) and 4.1-fold (2.3 μM) in the drug IC_50_ was observed in MCF-7 and T47-D cells, respectively, suggesting that the targeting of MK2 is a useful strategy to enhance killing of p53-defective cancer cells like T47-D [39,65,66].

#### 3.4.3. Evaluation of the Viability of Spheroids (3D Model)

**Figure 5:**
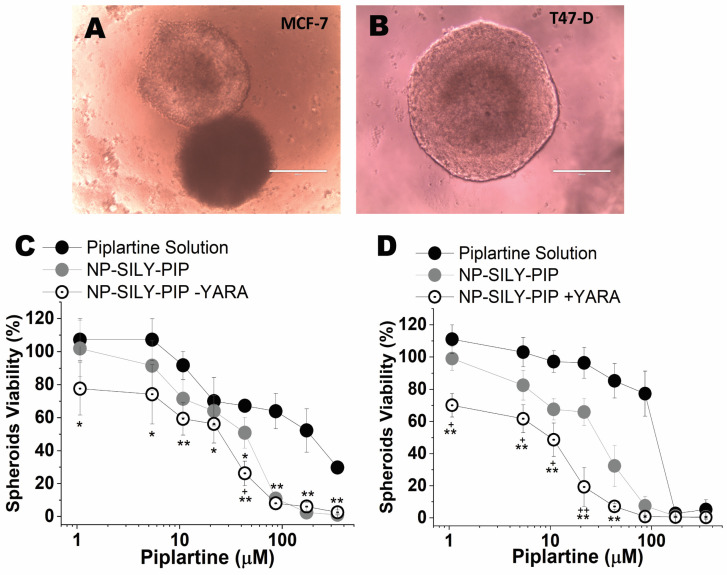
Formation and viability of spheroids after treatment with the nanoparticles. Sizes of MCF-7 (**A**) and T47-D (**B**) spheroids after 5 days in culture. Bar = 400 µm. Viability of the MCF-7 (**C**) and T47-D (**D**) spheroids after exposure to the drug solution in DMSO or to the nanoparticles with encapsulated piplartine or piplartine + YARA for 72 h. Data are shown as the average ± standard deviation of 12 spheroids in 4 independent experiments. * *p* < 0.05, ** *p <* 0.01 compared to the piplartine solution (DMSO) and + *p* < 0.05, ++ *p* < 0.01 compared to NP-SILY-PIP.

To bridge the gap between traditional 2D in vitro tests and animal models, we utilized a 3D culture approach with MCF-7 and T47-D cells. A three-dimensional (3D) culture is widely acknowledged as a more comprehensive and accurate method for conducting in vitro drug screening and formulation testing [67]. The capacity of spheroid models to replicate various elements of the in vivo environment, such as cell–cell interactions, hypoxia, drug penetration, resistance responses, and extracellular matrix production, has been well described [67,68]. Employing the liquid overlay technique, we successfully formed spheroids in approximately 91.0 ± 3.4% of wells. Inverted-phase contrast microscopy revealed that the spheroids from MCF-7 and T47-D cells displayed a spherical and uniform shape after five days of culture, as illustrated in Figure 5. The spheroids of the MCF-7 (Figure 5A) and T47-D (Figure 5B) cells had an average diameter of 770.3 µm ± 75.4 and 1033.8 µm ± 88.5, respectively.

The viability of spheroids was determined after 72 h of treatment with the drug in solution, unloaded NP-SILY, and nanoparticles containing only piplartine (NP-SILY-PIP) or piplartine + YARA (NP-SILY-PIP-YARA) (Figure 5C,D). As expected, the piplartine IC_50_ found in the 3D model was higher than in the monolayers, ranging from 27.5 µM to 27.9 µM for the T47-D and MCF-7 spheroids, respectively (Table 4). As observed for the cells in the monolayers, piplartine encapsulation in the nanoparticle increased the cytotoxic effect on the spheroids of both cell lines. Compared to the drug in solution, NP-SILY-PIP reduced the piplartine IC_50_ in MCF-7 spheroids by 4.9-fold (Figure 5C and Table 3). In T47-D spheroids, a similar result was obtained: NP-SILY-PIP reduced the drug IC_50_ by 4.6-fold (Figure 5D and Table 3). The presence of YARA contributed to a more pronounced effect in the nanoparticle cytotoxicity, promoting an even greater reduction in the spheroids’ viability compared to the drug solution. In the MCF-7 spheroids, this reduction was 8.0-fold (5.6 µM, *p* <0.001) and 15.6-fold (3.0 µM, *p* < 0.001) in the T47-D spheroids (Figure 5 and Table 4). The combination of drugs in the nanoparticles (NP-SILY-PIP-YARA) resulted in greater cytotoxicity in the T47-D spheroids compared to the MCF-7 spheroids. The difference in the piplartine IC_50_ between them was ~1.8-fold (Table 4), confirming the results obtained in the 2D cell culture.

### 3.5. Nanoparticle Uptake Studies

**Figure 6:**
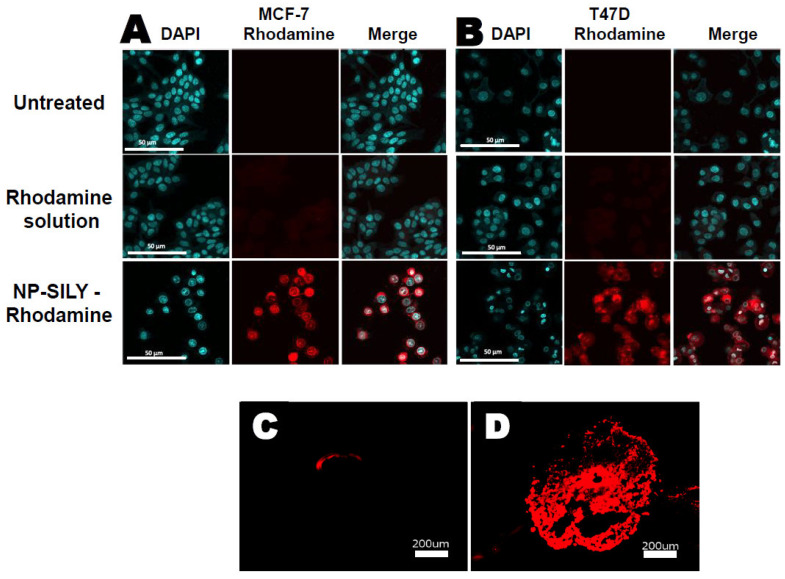
Uptake of rhodamine-labeled NP-SILY (50 μg/mL) and rhodamine solution (in DMSO) in cells as monolayers and spheroids after 48 h of incubation: (**A**) MCF-7 cells as monolayers; (**B**) T47-D cells as monolayers. The left images show nuclei highlighted in blue with Hoechst^®^ 33342 dye, the center images display Rhodamine-B, and the right images present a composite of the two previous images. (**C**) Rhodamine penetration in T47-D spheroids following treatment with rhodamine solution. (**D**) Rhodamine penetration in T47-D spheroids following treatment with rhodamine-labeled NP-SILY at a 50 μg/mL nanoparticle concentration. Scale bar = 200 µm.

Fluorescent-labeled nanoparticles were synthesized to investigate the nanoparticles’ cellular uptake. In our experimental design, we compared the cellular uptake of nanoparticles incorporating rhodamine within the polymer backbone to free rhodamine in solution. The incorporation of rhodamine into the nanoparticle’s shell allows us to track the nanoparticles. The nanoparticle uptake in the MCF-7 and T47-D cells (Figure 6A,B) was visualized by confocal microscopy following 48 h of incubation with rhodamine in solution and rhodamine-labeled nanoparticles. The penetration and distribution of free rhodamine and rhodamine-labeled nanoparticles were also evaluated in T47-D spheroids after 48 h of exposition (Figure 6C,D).

The treatment with rhodamine-labeled nanoparticles yielded strong fluorescent signals in both cell lines tested, highlighting the enhanced cellular uptake and penetration of the nanoparticles. This is in stark contrast to the more limited fluorescence observed with the rhodamine solution, indicating a substantially higher degree of nanoparticle penetration into cells compared to the limited cellular uptake of free rhodamine. The strong fluorescent signals from the rhodamine-labeled nanoparticles further support the effectiveness of our nanoparticles as delivery vehicles, capable of penetrating cellular barriers and releasing their cargo internally. The morphological changes and the decrease in cell numbers posttreatment align with our hypothesis that the nanoparticles are not only associated with the cells but also deliver their contents intracellularly.

A similar result was observed in the T47-D spheroids (Figure 6C,D). The T47-D cell line was selected as the spheroid model due to its robust and consistent spheroid-forming ability, which is crucial for replicable and uniform penetration assays. T47-D cells, derived from ductal breast carcinoma, exhibit a well-documented propensity to form cohesive and structurally stable spheroids that closely mimic the 3D architecture of solid tumors [69,70,71]. T47-D cells are known to form more tightly packed spheroids with a well-defined extracellular matrix, which can present a more challenging barrier for nanoparticle penetration [69,70,71].

Treatment with the rhodamine solution resulted in a smooth fluorescent rim surrounding the structure (Figure 6C), which indicates that the majority of the rhodamine was attached to the spheroid surface or penetrated the first cell layer. Conversely, the treatment with rhodamine-labeled nanoparticles (Figure 6D) resulted in a strong fluorescent signal into the core that appeared homogeneously throughout the spheroid. These results indicate that, unlike the drug in solution, the nanocarrier was able to pass the first cell layer and penetrate the deeper layers of the spheroid, which would enable the delivery of drugs more efficiently.

### 3.6. Retention of Nanoparticles in Mammary Tissue upon Intraductal Administration

**Figure 7:**
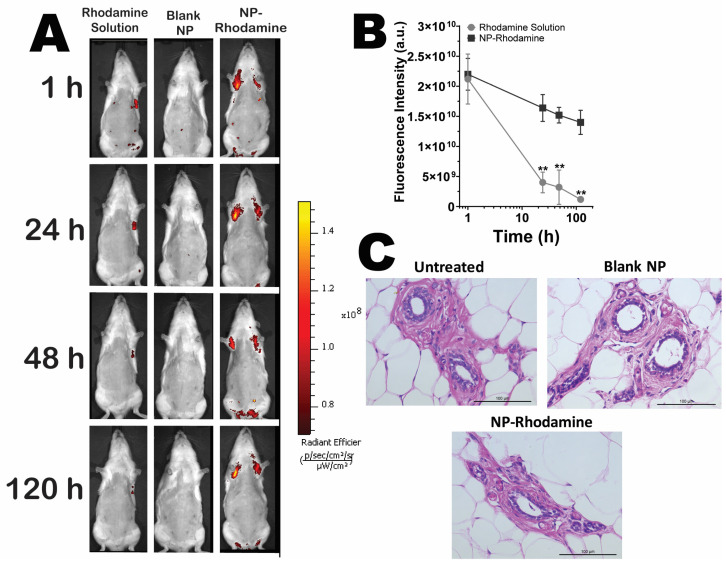
In vivo mammary tissue retention of the fluorescent marker rhodamine administered in nanoparticles or as a solution: (**A**) whole animal images showing fluorescence staining after intraductal administration of NP-Rhodamine or rhodamine solution (control); (**B**) mammary tissue fluorescence intensity decay as a function of time, *n* = 3 animals/group. ** *p* < 0.01 compared to NP-Rhodamine. (**C**) Histological sections of the mammary tissue of animals administered with blank NPs or NP-Rhodamine and compared with the untreated group. The pictures illustrate the structural integrity of ducts and the absence of inflammatory cell infiltration and edema. Scale bar = 100 μm.

In subsequent studies, the nanocarriers were evaluated for their ability to promote in vivo localization of fluorescent markers and induce histological changes in mammary tissue. Figure 7A shows representative images of animals subjected to intraductal administration of blank NPs (nonlabeled NPs), rhodamine-labeled nanoparticles, or rhodamine solution. Upon intraductal administration, the fluorescent marker solution demonstrated immediate drug distribution within the mammary tissue, comparable to that of NP-Rhodamine within the first hour. However, this initial localization was not sustained; fluorescence signals experienced a statistically significant attenuation (*p* < 0.01) after 24 h, indicating a rapid decline in the marker’s presence. Conversely, NP-Rhodamine exhibited a more durable localization, with detectable fluorescence persisting for up to 120 h postadministration, as evidenced in Figure 7A. This sustained visibility suggests that NP-Rhodamine provided a more stable retention profile for the therapeutic agent within the mammary tissue.

A quantitative assessment of the fluorescence intensity in the study subjects is presented in Figure 7B. Upon treatment with NP-Rhodamine, a more protracted and gradual diminution of the fluorescence signal was observed, contrasting with the more rapid dissipation seen with the drug in solution form. This suggests that while the route of administration facilitated localization to mammary tissue, it also led to a rapid clearance of rhodamine from the area. On the contrary, fluorescence from nanoparticles was consistently detectable, maintaining their presence for 120 h, underscoring the sustained retention capabilities of the NPs.

Figure 7C presents histological images of mammary tissue from both the control groups (untreated) and those treated with blank NPs or NP-Rhodamine. Consistent with prior descriptions, the untreated group exhibited mammary ducts lined by a single layer of cuboidal epithelial cells within a stromal matrix encircled by the fat pad [72]. Comparative analysis revealed that the mammary structures of animals treated with 1× PBS, blank NPs, and NP-Rhodamine are architecturally akin to those of the untreated controls. The absence of histopathological changes—such as edema, inflammatory cell infiltration, or ductal and lobular hypertrophy—indicates that the tissue’s structural integrity remained unaltered posttreatment.

### 3.7. Effectiveness of Nanoparticles in an In Vivo Carcinogenesis Model

**Figure 8:**
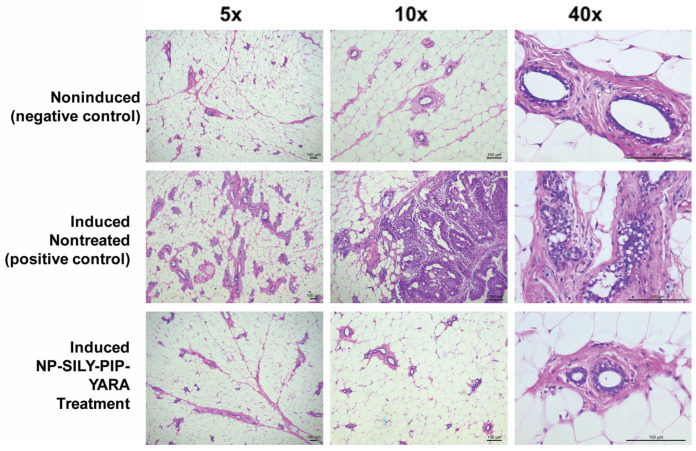
Effects of piplartine- and YARA-loaded NPs (NP-SILY-PIP-YARA) on the histological characteristics of breast tissue after MNU tumor induction compared to positive and negative control groups. A volume of 20 μL of the NP-SILY-PIP-YARA (1 mg/mL in 1× PBS) was administered per gland, and tissues were stained with H&E. Scale bar = 100 μm.

The purpose of this study was to assess the impact of administering NPs loaded with piplartine directly into the ducts on the development of mammary tumors in sexually mature female rats after MNU induction. The evaluation of MNU-induced mammary tumor growth in this study involved examining the following key factors (Table 5): frequency of tumors (i.e., incidence), number of tumors per animal (i.e., multiplicity), and size of these tumors [21]. Additionally, the study included histological analysis to examine the neoplastic formations (Figure 8).

Within the group subjected to NMU without subsequent treatment, 75% of the subjects exhibited detectable tumors at the 12-week mark following NMU exposure. The mean tumor count per subject was 1.83, with an average tumor volume of 28.3 mm^3^ (Table 5). These findings align with the established histopathological benchmarks associated with NMU-induced morphological changes [53,73]. In stark contrast, the treatment group that received NP-SILY-PIP-YARA exhibited a significantly reduced tumor incidence of only 14.2%, and the tumors that did develop were notably smaller in both count and volume, with an average tumor count of 0.14 per subject and a mean tumor volume of 4.6 mm^3^ (Table 5). In the noninduced control group, no tumors were developed, affirming the expectation of no spontaneous tumor formation without NMU induction and serving as an essential baseline for the study.

The examination of the mammary gland histology in the noninduced control group disclosed a normal tissue morphology, characterized by distinct architectural integrity and acinar structures embedded in connective tissue, as illustrated in Figure 8 Conversely, animals in the induced and nontreated groups exhibited signs of in situ carcinoma. This pathology was marked by significant lobe hyperplasia and the presence of dilated ducts overrun by neoplastic cells, with a concomitant reduction in connective tissue volume. Notably, a cribriform pattern of carcinoma involving the formation of intraductal bridges and microcalcifications was consistently observed across these specimens. Moreover, these carcinoma-afflicted tissues frequently displayed cytological atypia, including nuclear pleomorphism and increased mitotic activity, hallmarks of malignant transformation [74]. In contrast, the mammary gland architecture in the NP-SILY-PIP-YARA treated animals bore a close resemblance to that of the noninduced group, showing minimal or no evidence of hyperplasia (Figure 8). The glandular tissue in this treatment group maintained small, well-differentiated lobular structures.

The concentration of piplartine in the mammary tissue of animals that received NP-SILY-PIP-YARA was found to be 35.3 ± 22.4 μg/mL. The plasma concentration of piplartine was markedly lower, with a decrease of approximately 50-fold, quantified at 0.7 ± 0.05 μg/mL. This indicates a higher retention of piplartine in the mammary tissue, yet a portion of the piplartine was still absorbed into the bloodstream. No piplartine was detected in the mammary glands or plasma of the uninduced animal group, confirming that the quantification method was not influenced by any biological components.

## 4. Discussion

Polymeric nanocarriers represent a growing research area due to their unique properties and large potential in biomedical applications [75]. Here, we investigated the potential use of SILY-modified pNIPAm nanoparticles containing the combination of piplartine and YARA peptide to treat DCIS. Nanoparticles with a mean diameter of less than 400 nm were synthesized. The average mammary duct diameter ranges between 0.5 and 2 mm; thus, it is unlikely that the nanoparticles will pose a risk of ductal obstruction [17]. These nanoparticles present four main advantages, as discussed below.

The first advantage is the possibility of particle functionalization with SILY, a type I collagen-binding peptide. Given that collagen is a predominant component of the tumor microenvironment, and its content and structure are substantially altered by mutations in tumor suppressor genes in cancer cells, collagen affinity provides a strategic avenue for targeted therapy [10]. Consistent with earlier studies [43,76], we observed that nanoparticles modified with SILY successfully adhered to a collagenous matrix, a property not observed in nonmodified nanoparticles. In line with this finding, the nanoparticles provided longer retention in vivo in the mammary tissue compared to a simple solution. This finding is particularly important considering collagen’s established role as a key component of the stromal tissue around breast ducts, where it has been shown to play a vital role in both the onset and spread of tumors (i.e., metastasis) [77,78]. This could have significant implications for the development of more effective and targeted cancer treatments, particularly in tumors in which collagen alterations are a prominent feature.

The second advantage is the observed temperature-responsive behavior of the nanoparticles, particularly in relation to their lower critical solution temperature (LCST), which is crucial for drug delivery [79]. Below the LCST, nanoparticles remain in a swollen state, ideal for absorbing therapeutic agents, while above the LCST, they shrink, facilitating drug release at physiological or locally elevated temperatures. While the incorporation of hydrophobic drugs like piplartine lowers the LCST, the phase transition persists allowing for control over drug release [80]. In fact, drug encapsulation increased the hydrophobic collapse of poly(N-isopropylacrylamide) nanoparticles by masking the molecule’s natural charge, which reduces its solubility in polar solvents like water [81].

A third advantage is that the nanoparticles enabled co-encapsulation and controlled release of piplartine and YARA. The addition of AMPS to the nanoparticle synthesis maximizes the loading of the cationic peptide YARA via electrostatic interactions [31,57,82]. The resulting nanoparticles displayed a negative zeta potential after core removal, confirming the retention of an anionic charge and indicating stable particle formation, consistent with prior research [27,57]. The hollow or low polymer density, which reduced the diffusion barrier compared to solid nanoparticles, coupled with the anionic nature of the nanoparticles contributed to a high drug-loading capacity for both piplartine and YARA [31]. Additionally, modification with SILY significantly increased the YARA-loading efficiency, which could be attributed to the peptide’s influence on the spatial orientation or distribution of charges on the nanoparticle surface, creating a more conducive environment for YARA loading, possibly through enhanced electrostatic interactions or surface hydrophilicity.

In the context of diseases like cancer, prevalent homeostatic chemical imbalances, including increased enzymatic activity, acidic pH shifts, altered redox states, or a rise in reactive oxygen species [83], are useful for developing and refining therapies. Salts and additives in the surrounding medium influence drug release through osmolality variations, and contribute to discrepancies between in vitro and in vivo drug release rates, with in vivo conditions typically accelerating polymer degradation and drug release [84]. To emulate in vivo conditions more closely, degradation and drug release studies were conducted at pH 7.4 and 3.5. The introduction of reducible disulfide crosslinks in the nanoparticles was a strategic measure to prevent drug entrapment and ensure effective degradation and release. While nanoparticles in PBS at pH 7.4 showed minimal degradation over 48 h, exposure to pH 3.5 initiated nanoparticle fragmentation over time, a process that could be facilitated by the reduction in disulfide bonds in the presence of intracellular glutathione and the acidic environment of the late endosome/lysosome compartments, leading to nanoparticle degradation and drug release following cellular uptake [85,86].

The crosslinking with BAC enhanced the degradation of nanoparticles, which is a crucial factor for both effective drug delivery and safety. The degradation process is particularly relevant in the intracellular environment, where disulfide bonds can be cleaved in the presence of higher concentrations of glutathione, a reducing agent found in cells [27,30]. This intracellular environment also typically exhibits a different pH compared to extracellular spaces. By focusing on pH, we were able to explore how these nanoparticles behave in the unique pH conditions of cancer cells, which is vital for ensuring that the drug is released inside the cells where it is most needed [24,25]. Furthermore, the degradation of nanoparticles is not only essential for the release of the therapeutic agents but also for the subsequent elimination of the nanoparticles from the body, addressing safety concerns. While the thermoresponsive nature of pNIPAM is intriguing, our study aimed to first establish a foundational understanding of how pH and disulfide bond cleavage within the cellular environment influence the behavior of these nanoparticles, considering both therapeutic efficacy and safety. However, previous studies have already demonstrated a dual stimuli-triggered drug release from pNIPAM systems in which increased temperature, in addition to changes in pH, promoted early drug release due to the shrinkage observed in the PNIPAM backbone [87]. Thus, future research could, indeed, explore the additional layer of temperature sensitivity to create a more comprehensive drug delivery system.

The nanoparticles demonstrated release of both drugs over a 5-day period, attributed to their high loading efficiency and continuous degradation of the nanoparticle disulfide crosslinkers. Degradation studies revealed that the final release rate of the drugs was significantly higher at pH 3.5, with an approximate 22% increase for both piplartine and YARA compared to pH 7.4. The enhanced release in acidic conditions aligns with the expected behavior of disulfide crosslinkers, which are known to be more susceptible to reduction and subsequent breakdown in acidic environments [27]. The absence of a dense core in these nanoparticles facilitates drug diffusion and reduces the likelihood of permanent drug entrapment. As a result, these structural and interactive properties of the nanoparticles contribute to an increased and sustained release rate, ensuring effective delivery of piplartine and YARA to the target site for enhanced cytotoxicity against cancer cells.

The quick release of electrostatically bound drugs could raise a valid concern about the potential for nonspecific binding of the drug to cell membranes. This is a common issue in nanoparticle-based drug delivery, where the initial burst release often results from the surface-bound drug being released rapidly before the drug encapsulated within the nanoparticle matrix [88]. Nevertheless, the phenomenon of multiphase drug release in nanoparticle-based drug delivery systems is widely recognized. After the initial burst release, a sustained release phase occurs, which can be attributed to the gradual degradation of the nanoparticle matrix, the diffusion of the drug from the core of the nanoparticles, or a combination of both [89,90]. The pNIPAM nanoparticles, especially when modified with crosslinkers like BAC, may not completely dissociate immediately upon a temperature change, and the reduction in size could indicate a partial collapse or densification of the nanoparticle structure rather than complete dissociation [91,92]. As the matrix slowly degrades or dissolves over time, it can continue to the release of the encapsulated drug. In addition, it should be noted that the nanoparticles were designed for local intraductal delivery. Thus, even if a part of the drug content was released within 12 h, it would be present at the site of action, limiting systemic effects.

Another advantage of the nanoparticles is the improved cytotoxic effects of piplartine, represented by a lower IC_50_, while the nanoparticle, per se (i.e., unloaded), exhibited negligible cytotoxicity within the tested concentration range, aligning with prior studies that have established the biocompatibility of pNIPAM nanoparticles [30,57,93]. The nanoparticles were found to augment the cytotoxicity of piplartine similarly in both the MCF-7 and T47-D cell lines. Increases in drug cytotoxicity mediated by nanoencapsulation has been demonstrated in other studies [21,94,95]. Notably, a more substantial decrease in the IC_50_ of piplartine was observed upon the co-encapsulation of YARA within the nanoparticles, which is congruent with previous research demonstrating that the inhibition of MK2, a known cell survival factor in conjunction with mitomycin-c treatment, leads to reduced cell viability [96]. Similarly, an increased cytotoxic effect of cisplatin and doxorubicin has been reported when used in conjunction with MK2 inhibitors [65,97]. The efficacy of nanoparticles containing YARA in reducing the nuclear migration of pSmad3 and the phosphorylation of HSP27 ser15, both integral components of the MAPK signaling pathway, was also evident. These outcomes provide substantial evidence that the strategic co-encapsulation of YARA and piplartine within the nanoparticles plays a critical role in heightening their cytotoxic impact. This underscores the additive enhancement afforded by this combinational approach in the realm of nanoparticle-mediated cancer therapy.

The IC_50_ values obtained in the spheroids were higher than those obtained in monoculture, which was previously related to the limitation of drug penetration into the spheroid core [76]. Consistent with the findings observed in monolayer cultures, nanoencapsulation markedly reduced the piplartine IC_50_ across both examined cell line spheroids. Analogously, the administration of the drug combination intensified the piplartine cytotoxic impact in the MCF-7 spheroids. This effect was further amplified in the T47-D spheroids. As described above, emerging research on the involvement of MK2 in the DNA damage response, especially its synthetic lethal interaction following genotoxic stress in cells lacking functional p53, suggests that the absence of functional p53 in the T47-D cell line could have enhanced the response to the cytotoxicity induced by the drug combination encapsulated in nanoparticles [38]. Together, the data support integrating piplartine and YARA into the nanoparticles to enhance the efficacy of the nanocarrier system, especially when p53 mutations exist.

The observed enhancement in cytotoxicity with nanoparticle delivery aligns with the findings of an increased cellular uptake in both individual cell cultures and spheroid models. This suggests that the cells, whether grown in a flat, two-dimensional culture or in three-dimensional spheroid form, more effectively internalize the encapsulated agents, leading to heightened toxicity. The thermoresponsive nature of poly(N-isopropylacrylamide) (pNIPAM) plays a key role in enhancing cellular uptake. It has been suggested that when pNIPAM reaches its lower critical solution temperature (LCST), it undergoes a phase transition from a hydrophilic to a hydrophobic state, resulting in greater interaction with cell membranes and, consequently, greater cellular uptake [98]. Additionally, the size reduction of pNIPAM nanoparticles at body temperature might make them more suitable for endocytosis, the primary mechanism for cellular internalization of nanoparticles. While Figure 6 does not directly show particle uptake, in a recent work by our group, it was observed that SILY-modified PNIPAM nanoparticles undergo clathrin-mediated endocytosis by breast cancer cells; lipid nanoparticles coated with SILY underwent endocytosis mediated by both clathrin and caveolin, suggesting a greater influence of the composition of the nanoparticle itself than that of the SILY coating [76]. Nevertheless, SILY molecules, when attached to the surface of nanoparticles, can specifically bind to collagen, which is abundant in the extracellular matrix of tissues and facilitates the localization of the nanoparticles near the target cells, potentially increasing the likelihood of cellular uptake. However, whether this increases the actual internalization of the nanoparticles by cells depends on several factors, including the density of SILY on the nanoparticle surface and the specific cell type [99,100].

Ultimately, the effectiveness of the nanoparticle system was validated through in vivo studies. In the design of this experimental framework for the study of nanoparticle efficacy in the MNU animal model, the selection of control groups was critical to ensure the clarity and relevance of the results obtained. The induced nontreated animals served as the positive control to establish the baseline tumor development post-NMU administration. Uninduced animals were included as a negative control to confirm the absence of spontaneous tumor formation. The therapeutic intervention was represented by the NP-SILY-PIP-YARA treatment group, which contained active pharmaceutical agents and represented the most potent construct in vitro. The decision to omit blank nanoparticles from the tumor induction experiment was informed by prior observations where blank nanoparticles, structurally similar nanoparticles without the therapeutic payload, displayed no cytotoxicity in the cell culture and in the histologic analysis. These results suggest that the blank nanoparticles did not exert a biological effect on tumor development or progression. By focusing on the comparison between the positive and negative controls and the treatment group, the study aimed to yield unambiguous data on the antitumor efficacy of the NP-SILY-PIP-YARA formulation.

The stark reduction in both tumor incidence and size in the NP-SILY-PIP-YARA treated group, as compared to the untreated NMU-exposed group, suggests a potent inhibitory effect of the treatment on NMU-induced mammary tumor development. The absence of tumor development in the noninduced group further substantiates the specificity of NMU in inducing tumors and the efficacy of NP-SILY-PIP-YARA in mitigating this effect. In contrasting to the positive control group, the NP-SILY-PIP-YARA treated group exhibited mammary gland histology that paralleled the noninduced group, with minimal to no signs of hyperplastic activity. The lobular structures in these specimens were small and well differentiated, indicative of normal cellular differentiation and a positive therapeutic response. This pronounced histological difference highlights the potential of NP-SILY-PIP-YARA treatment in maintaining mammary tissue integrity and preventing carcinogenic progression.

Notably, the concentration of piplartine within mammary glands treated with nanoparticle formulations was significantly higher than that in plasma, showing a local retention of the drug. Previously, our group developed and evaluated a nanoemulsion for the intraductal delivery of piplartine in the same model of breast carcinoma [21]. Compared to the nanoemulsions, the pNIPAM nanoparticles presented several advantages. Although the nanoemulsion promoted mammary localization of the drug and piplartine levels in breast tissue were 18-times higher than in plasma, the pNIPAM nanoparticle developed in the present study was able to further increase the tissue localization of the drug and led to a 50-fold greater amount of drug in breast tissue compared to the plasma. This may be related to the unique aspects of the pNIPAM nanoparticle developed in your study, particularly the surface modification with SILY and collagen binding. Another advantage of the pNIPAM nanoparticle is the co-encapsulation of two drugs, offering a multifaceted approach to therapy. Moreover, the nanoparticles exhibit responsive behavior to environmental stimuli (like temperature), which can be finely tuned for targeted drug release. The transition from a nanoemulsion-based delivery system to a more advanced nanoparticle system represents strategic evolution in drug delivery research. While nanoemulsions are effective, the pNIPAM nanoparticles provide a more controlled release mechanism, essential for drugs that require precise dosing to achieve optimal therapeutic outcomes.

## 5. Conclusions

The present study elucidates the potential of polymeric nanocarriers, specifically SILY-modified pNIPAm nanoparticles encapsulating piplartine and YARA peptide, for the localized treatment of DCIS. The synthesized nanoparticles, with their sub-400 nm diameter, could avoid the risk of ductal obstruction while ensuring efficient cellular permeability and drug absorption. The nanoparticles were demonstrated to be an effective platform for the loading and delivery of the chemotherapeutic agent piplartine and the peptide YARA, with a high loading capacity and prolonged drug delivery profiles. Their internalization in both two-dimensional and three-dimensional cell cultures, confirmed by confocal microscopy, underscores their superior cellular penetration capabilities. The temperature-responsive behavior of these nanoparticles is pivotal for drug loading and controlled drug release. The in vitro and in vivo studies validate the biocompatibility of unloaded nanoparticles and their augmented cytotoxicity when loaded with piplartine and YARA, especially against cancer cells. The enhanced cytotoxic effects observed in the spheroid cultures and the differential responses in both cell lines further affirm the efficacy of this nanoparticle system in cancer therapy. Overall, this study demonstrates the significant potential of NP-SILY-PIP-YARA nanoparticles in targeted cancer therapy, leveraging their unique properties for efficient drug delivery, reduced systemic exposure, and enhanced therapeutic efficacy. This approach could pave the way for more effective and targeted cancer treatments, particularly in tumors in which collagen alterations are a prominent feature.

## Figures and Tables

**Table 1:** Size, PDI, and ζ potential of nanoparticles. The size represents the z-average diameter of measured nanoparticles at 25 °C. NP-PIP-YARA: NPs containing piplartine and the peptide YARA; NP-SILY-PIP-YARA: SILY-modified NPs containing piplartine and YARA.

Nanocarrier	Size (nm)	PDI	Zeta Potential (mV)
Unloaded NPs	370.7 ± 11.5	0.15 ± 0.007	−31.3 ± 8.4
NP-PIP-YARA	380.6 ± 13.4	0.21 ± 0.06	−25.9 ± 2.05
NP-SILY-PIP-YARA	387.1 ± 12.8	0.22 ± 0.02	−8.9 ± 0.38

**Table 2:** Drug loading: data show the amount of drug loaded per milligram of nanoparticle and represent the mean ± standard deviation of 12 replicates obtained in 4 independent experiments.

Nanocarrier	YARA Loading (mg/mg)	Piplartine Loading (mg/mg)
Non-modified NPs	0.753 ± 0.05	0.921 ± 0.30
NP-SILY	1.262 ± 0.10	0.733 ± 0.04

**Table 3:** Influence of piplartine nanoparticle encapsulation and co-encapsulation with YARA on piplartine IC_50_ values and 95% confidence interval (CI) values.

Treatment	Cell Line and IC_50_ (μM)		95% CI (µM)	
	MCF-7	T47-D	MCF-7	T47-D
Piplartine solution	10.6	9.5	5.2–20.6	4.9–15.8
NP-SILY-PIP	6.6	6.1	3.2–12.0	4.1–10.4
NP-SILY-PIP-YARA	4.0	2.3	2.2–8.0	0.7–3.4

**Table 4:** Influence of piplartine encapsulation in NPs and its co-encapsulation with YARA on the IC_50_ with 95% confidence intervals for piplartine in MCF-7 and T47-D spheroids.

Treatment	Cell Line and IC_50_ (μM)		95% CI (µM)	
	MCF-7	T47-D	MCF-7	T47-D
Piplartine solution	133.8	104.8	113.4–158.1	84.7–130.0
NP-SILY-PIP	27.2	22.7	25.5–34.3	19.9–25.6
NP-SILY-PIP-YARA	16.6	6.7	14.1–19.4	5.7–7.7

**Table 5:** Tumor parameters for the NP-SILY-PIP-YARA group compared to the untreated (positive control) and uninduced (negative control) groups. The results are the mean ± standard deviation of 8 animals per group.

Group	Induced Nontreated	NP-SILY-PIP-YARA	Control Uninduced
Incidence (%)	75	14.2	0.0
Multiplicity	1.83 ± 0.9	0.14 ± 0.3	0.0
Size (mm^3^)	28.3 ± 8.2	4.6 ± 1.8	-

## Data Availability

Data are contained within the article.
